# Illumina Short-Read Sequencing of the Mitogenomes of Novel *Scarites subterraneus* Isolates Allows for Taxonomic Refinement of the Genus *Scarites* Fabricius 1775, within the Carabidae Family

**DOI:** 10.3390/insects13020190

**Published:** 2022-02-11

**Authors:** Elliot C. Kyndt, John A. Kyndt

**Affiliations:** College of Science and Technology, Bellevue University, Bellevue, NE 68005, USA; ekyndt04@gmail.com

**Keywords:** *Scarites*, *nebraskensis*, arkansensis, Carabidae, Illumina, short-read sequencing, mitochondrial genome, taxonomy, Cox1, 18S rRNA

## Abstract

**Simple Summary:**

Ground beetles (Carabidae) have important ecological functions and serve as food, biological pest control, and models in biological research. Although there are over 40,000 ground beetle species worldwide, only a small fraction of those has genomic information currently available, which limits their classification and understanding of diversity. Since next-generation genome sequencing has become more mainstream, we used Illumina short-read sequencing to obtain complete mitogenomes from two *Scarites* beetles that we collected ourselves in Nebraska and Arkansas. *Scarites* are large ground beetles that resemble tropical beetles with a big head and large mandibles, and their role as predator and prey helps maintain sustainability in local ecosystems. This straightforward sequencing and analysis was found to be accurate and sufficient to help classify these isolates to the subspecies level. This is the first report of mitogenomes for *Scarites subterraneus* and only the second in that genus. This method is easily applicable to more beetle species and can be used to increase our understanding of beetles worldwide.

**Abstract:**

We sequenced the complete mitogenomes, 18S and 28S rRNA of two new *Scarites* isolates, collected in Eastern Nebraska and Northern Arkansas (US). Based on molecular sequence data comparison and morphological characteristics, the new isolates were identified as a subspecies of *Scarites subterraneus* Fabricius 1775, for which we propose the subspecies names ‘*nebraskensis*’ and ‘*arkansensis*’. The new 18S and 28S rRNA sequences were found to be 99% and 98% identical to *Scarites subterraneus*. There are no other *Scarites* 18S or 28S rRNA sequences in the Genbank database, however, phylogenetic analysis of the Cox1 genes showed *S. vicinus* Chaudoir, 1843, and *S. aterrimus* Morawitz, 1863, as the closest relatives. This is the first report of a mitogenome for *S. subterraneus*, and only the second mitogenome for that genus. The nucleotide sequence identity between the mitogenomes of the two isolates is 98.8%, while the earlier sequenced *S. buparius* Forster 1771 mitogenome is more distantly related, with only 90% (to ssp. *nebraskensis*) and 89% (to ssp. *arkansensis*) overall nucleotide sequence identity. These new mitogenomes, and their phylogenetic analysis, firmly establish the position of *Scarites* on the Carabidae family tree and further refine the genus. In addition to the molecular data provided for the *Scarites* species, this approach also allowed us to identify bacterial and viral signatures for *Providencia*, *Myroides*, *Spiroplasma*, and a giant *Nucleocytoviricota* virus, associated with the *Scarites* species. We hereby present a simple and efficient protocol for identification and phylogenetic analysis of *Scarites*, that is applicable to other Coleoptera, based on total DNA extraction and Illumina short-read Next-Gen sequencing.

## 1. Introduction

*Scarites* is a genus of big-headed ground beetles that belong to the family Carabidae (Coleoptera), which is one of the largest families of beetles [1,2]. The Carabidae is one of the dominant groups of terrestrial predators that is found worldwide, with around 40,000 species, and about 190 species of *Scarites* have been found that are native to the Paleartic, the Near East, North America, and North Africa [3]. There are seven *Scarites* species found in North America [4,5]. *Scarites* are large (14–30 mm), almost like tropical stag beetles, but they do not share any ancestry, and have large mandibles and black shiny bodies. An interesting behavioral feature is that when they are touched, they will play dead, folding their legs in and sticking their head up, thereby often avoiding predation (personal observation). *Scarites* are fierce predators themselves, both as larva and adult, and are often found under bricks, wood logs, mulch, or beneath rocks lining garden flower beds. With their distinctive large mandibles, they hunt and kill a wide variety of pests including snails, slugs, grubs, ants, and soil dwelling caterpillars such as cutworms and armyworms [6]. *Scarites* are often described as beneficial to gardens as they enhance sustainability of ecosystems by reducing pest populations [7,8]. 

The genus *Scarites* is currently composed of 12 named species: *acutidens*, *aterrimus*, *buparius*, *cayennensis*, *eurytus*, *hespericus*, *laevigatus*, *occidentalis*, *quadriceps*, *subterraneus*, *terricola*, *vicinus* (as retrieved from NCBI Taxonomy, 2021). The two most widespread members (and the only two found in Nebraska) are *S. vicinus* and *S. subterraneus*. *S. subterraneus* was originally described by Fabricius in 1775, with a revised description by Bousquet and Skelley [9]. *S. texanus* was synonymized under *S. subterraneus*, while *S. vicinus*, which was previously considered a junior synonym of *S. quadriceps*, was revalidated. *Scarites subterraneus* are mostly found in the Caribbean Sea, Central and North America. 

Despite the critical role beetles play in most terrestrial ecosystems [7,10,11,12], the molecular data available, especially genomic data, is still limited compared to many other taxa [13,14], which impedes the development of a full phylogenetic analysis in many genera. Even though the family of Carabidae is the dominant group of Adephaga found worldwide [15], the taxonomy is often challenging, especially at the species level, mainly because it suffers from the lack of molecular data from enough species to support the documented morphological characterization. Classification often relies on morphological features or single gene-based (mainly 18SrRNA) or recently on specific genomic probe comparisons [16,17,18,19]. These methods have proven valuable at the family and genus level, but do not contain the higher resolution necessary for species (and sometimes even genus) level characterization. Despite the 40,000 or so Carabidae species, only five whole genomes and only 80 mitochondrial genomes are currently found in NCBI Genbank (of which 13 are unverified, indicating that there are potential inconsistencies and possible genome annotation problems in them). This limited number of genomes available limits the potential for in-depth molecular phylogenetic studies. 

The taxonomy of North American *Scarites* is based on morphological descriptions that contains several uncertainties, and is therefore considered provisional, awaiting further refinement from DNA sequence analysis [9]. Unfortunately, there is only limited molecular data available for *Scarites*, and for the *Scaritinae* subfamily in general. Molecular DNA-based phylogenetic studies typically rely on ribosomal RNA sequences or mitogenomes (or a combination thereof) [16,17,20,21,22]. However, the use of these in taxonomic placement of the *Scarites* species has been hampered due to the lack of available data. Only one 18S rRNA gene and a partial 28S rRNA gene for *S. subterraneus*, and one partial mitogenome for *S. buparius*, were available at the start of this study. Partial or complete Cox1 gene sequences were found for 18 *Scarites* species or subspecies in the database, however, most of these lack a detailed published species identification, and a phylogenetic analysis using this gene has not been published for the *Scarites* genus. We set out to characterize and perform a phylogenetic comparison of *Scarites* species that we collected from Eastern Nebraska (US) and Northern Arkansas (US), using both morphological characterization and molecular sequencing data obtained from Illumina short-read NGS. 

## 2. Materials and Methods

### 2.1. Species Isolation

The first *Scarites* species used in this study were isolated in Omaha, Nebraska (41°15′18″ N; 96°06′55″ W) in July 2020 and were found underneath bricks that are part of an unused firepit. Three specimens with a body length of 19–20.5 mm were preserved in 80% ethanol and stored immediately in a −20 °C freezer for at least 12 months, after which they were further used in this study. The second set of specimens were found at the Tanyard Creek area in Northern Arkansas (near Bentonville; 36°28′12.378″ N; 94°15′33.51″ W) in October 2021. Three specimens with a body length of 18.5–19.5 mm were found under large rocks in a forest area. They were preserved in 80% ethanol in a −20 °C freezer for 2 months. Determination the sex in *Scarites* species is challenging because the protarsi do not exhibit the sexual dimorphisms typical of other carabid genera (the key can be found at: https://bugguide.net/node/view/2990, accessed on 24 December 2021). Since genitalia were hidden in our specimen, we could not come to a conclusive determination of the sex in the used preserved specimen. Images were taken using a 10× and 20× magnification on a SWIFT Optical SM100 microscope. Measurement and scale were determined using a Ward’s stage micrometer. 

### 2.2. DNA Extraction

Three preserved *Scarites* specimens from each location were combined for total DNA extraction. Specimens had been preserved in 80% ethanol, and the gut insides were removed from the abdomen of each using a sterile scalpel and tweezers. After removal of the guts, the specimens were rinsed in 70% ethanol and ground up with a sterile mortar and pestle into a fine powder. In total, 220 mg of ground *Scarites* powder from the Nebraska specimens and 120 mg of the Arkansas specimens was used for total DNA extraction using the FastDNA Spin Kit from MPBiomedicals. DNA analysis using Qubit and NanoDrop showed a DNA concentration of 254 ng/μL for the Nebraska samples and 172 ng/μL for the Arkansas samples, with a 260/280 nm absorbance ratio of 1.74 (Nebraska sample) and 1.81 (Arkansas sample). A total of 300 ng of DNA from each sample was used for DNA sequencing.

### 2.3. DNA Sequencing and Annotation

The sequencing library was prepared using the Illumina Nextera DNA Flex Library Prep kit. The genomes were sequenced by an Illumina MiniSeq, using 500 μL of a 1.8 pM library. Paired-end (2 × 150 bp) sequencing generated 637,944 reads and 70.3 Mbps for the Nebraska sample and 1,747,866 reads and 263.9 Mbps for the Arkansas sample. In both cases, the sequence read length distribution was 35–151 with >90% of the read lengths above 149 bp. Quality control of the reads was performed using FastQC [23] within Basespace (Illumina; version 1.0.0), using a k-mer size of 5 and contamination filtering. We assembled the genome de novo using SPAdes (version 3.9.0; [24]) and Velvet (version 1.2.10; [25]) within BaseSpace (Illumina) and Unicycler (version 0.4.8; [26]) within the PATRIC resource server [27]. The SPAdes contig comprising the mitogenome for each species was annotated using MITOS2 (MITOS WebServer: http://mitos2.bioinf.uni-leipzig.de/index.py; accessed on 24 December 2021 [28,29]). Some of the coding regions were manually refined using BLAST comparison with reference published mitogenomes (NCBI Genbank, and [30]).

Primers for 18S and 28S rRNA amplification were designed by us and synthesized by Sigma Aldrich. Sequences were as follows: ScarEK_18S_F: 5′ TCATATGCTGTCTCAAAGATTAAGC 3′; ScarEK_18S_R: 5′ CTTAAGTTTGTCTTGCGACGATCC 3; ScarEK_28S_F: 5′GATTCCCTAAGTAGCGGCGAGC 3′; ScarEK_28S_R: 5′ CAGCATGAACGCTCTTAGTGCG 3′. Amplified fragments were gel-purified using the PureLink Quick Gel Extraction and PCR purification kit (Invitrogen) and sequenced by Sanger sequencing using the respective primers (at the Iowa State University DNA core facility). Forward and reverse fragments were aligned using ClustalW and combined into a single 18S and 28S fragment. These fragments were aligned with the Illumina NGS assembled contigs using Clustal Omega [31].

Taxonomic classification of the assembled contigs was performed with Kraken2 [32] within PATRIC [24]. All database genomes were used for this taxonomic classification. The phylogenetic charts were generated by Krona [33]. 

### 2.4. Phylogenetic Trees and ANI Calculations

The alignments for the mitogenome, Cox1 gene, and 18S rRNA comparisons were performed using Clustal Omega [28]. The evolutionary history was inferred by using the Maximum Likelihood method and General Time Reversible model [34]. Evolutionary analyses were conducted in MEGA X [35]. Initial tree(s) for the heuristic search were obtained automatically by applying Neighbor-Join and BioNJ algorithms to a matrix of pairwise distances estimated using the Maximum Composite Likelihood (MCL) approach, and then selecting the topology with superior log likelihood value. A discrete Gamma distribution was used to model evolutionary rate differences among sites (5 categories (+G, parameter = 0.2185)). The bootstrap consensus values were inferred from 500 replicates [36]. The trees are drawn to scale, with branch lengths measured in the number of substitutions per site. iTOL was used to draw the phylogenetic trees expressed in the Newick phylogenetic tree format [37]. Average percentage nucleotide identity between the genomes was calculated using Jspecies [38].

## 3. Results and Discussion

### 3.1. Species Identification Based on Morphology

**Figure 1:**
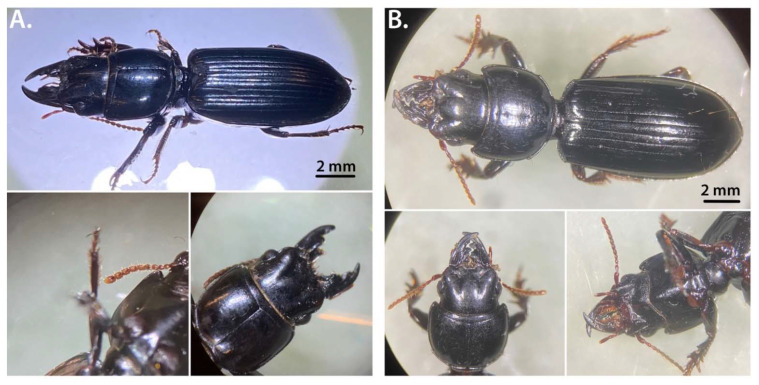
Images of the newly isolated *Scarites* specimen used in this study. (**A**) shows the full *Scarites* sp. *Nebraskensis* species, and detailed images of the antennomeres, thorax and mandibles. (**B**) shows the same details of the *Scarites* sp. *Arkansensis* species.

Figure 1 shows representative images of the specimen used in this study. Figure 1A contains images of the Nebraska isolates specimens, while Figure 1B is from the Arkansas isolates. The larger morphological features clearly show that the specimen belongs to the *Scarites* genus, which is a genus of ground beetles that is native to North America and North Africa [3]. For the initial species identification, based on morphological features, we used a key designed after Bousquet and Skelley [9] and Nichols [39], with modifications and expansions by Peter W. Messer (the key can be found at: https://bugguide.net/node/view/2990, accessed on 24 December 2021). As mentioned in the key description, this provisional taxonomy of North American *Scarites* awaits refinement from future morphological scrutiny and DNA sequence analysis.

Thus far, only two *Scarites* species have been described in Nebraska: *vicinus* (belonging to the *quadriceps* group) and *subterraneus*. To distinguish between the *quadriceps* and *subterraneus* group, one compares the average body length, elytron length, and antennomeres length/width. The specimen used in this study had an average body length of 19.8 mm and average elytron length of 10 mm. According to the key, an average body length ≤20.5 mm and elytron length ≤10 mm places the species in the *subterraneus* group. In addition, the antennomeres 8–10 do not appear to be longer than wide, and there is no obvious elongation of segments 5–7 (moniliform) (Figure 1), which is also consistent with a *subterraneus* species placement. 

An additional distinguishing factor comes from comparing the seams in the thorax. In Figure 1, a distinct “V” in the bottom part of the central line on the thorax can be observed. In *S. vicinus* and *quadriceps*, this is a straight line. To tell the difference between the latter two species, one looks at the lobes in the head and proportions of head to thorax. *S. vicinus* tends to have the rounder thorax with head and thorax being nearly equal in volume (with mandibles) whereas *S. quadriceps* and *S. subterraneus* have three lobed heads.

As stated before, *S. subterraneus* has the pronounced “V” in the thorax seams. The above analysis indicates that the specimens we isolated from these locations both presumably belong to the species *S. subterraneus*, based on morphological feature comparison, however, to further confirm their taxonomic placement, molecular genetics and genomics data was necessary. 

### 3.2. Genome Assembly

After Illumina paired-end sequencing, a total of 637,944 reads and 70.3 Mbps was obtained for the Nebraska sample, and 1,747,866 reads and 263.9 Mbps for the Arkansas sample. We attempted de novo assembly with three different programs, SPAdes, Unicycler, and Velvet. For the Nebraska specimen, the SPAdes assembly yielded 336 contigs (>1000 bp) and was 601,914 bp in length. The largest contig was 16,241 bp in length. The Unicycler assembly yielded 39 contigs (>1000 bp) and total length of 108,015 bp, while the Velvet assembly yielded 9 contigs (>1000 bp) with only a total length of 49,497 bp. The largest Unicycler and Velvet contigs were 16,164 and 15,322 bp, respectively. After an initial NCBI BLAST of the larger contig of these datasets, it became clear that all three assemblies consisted of mitochondrial DNA that showed 90% identity to the *Scarites buparius* mtDNA in the database. The mtDNA assemblies were identical to each other, but since the SPAdes 16.2 Kbp assembly was larger, we continued with that for further analysis. 

For the Arkansas species, the SPAdes assembly yielded 2688 contigs (>1000 bp) and was 4,154,694 bp in length, with the largest contig 16,240 bp. The Unicycler assembly yielded 44 contigs (>1000 bp) and a total length of 955,504 bp, while the Velvet assembly yielded 17 contigs (>1000 bp) with only a total length of 56,463 bp. The largest Unicycler contig was 16,163 bp, however, the largest Velvet contig was only 3028 bp. Like the Nebraska species assembly, the SPAdes 16.2 Kbp contig was found to be the mitogenomic DNA of *Scarites* and was used for further analysis. 

In addition to the larger contig, we also analyzed the smaller contigs and identified an 18S rRNA sequence as part of a 3014 bp contig, and a 28S rRNA sequence in a 5447 bp contig, both obtained from the Nebraska Velvet assembly. The 18S rRNA for the Arkansas species was found on a 3251 bp contig in the Unicycler assembly. When performing an NCBI BLAST we found both 18S rRNA sequences to be 99% identical to the *Scarites subterraneus* 18S rRNA (1997/2004 bp; [16]) and the 28S rRNA to be 98% identical to *Scarites subterraneus* (1238/1262 bp; partial gene sequence). There are no other *Scarites* 18S or 28S rRNA sequences in the Genbank database, and the closest relative in the analysis was *Pogonus iridipennis* with 87% identity for the 18S rRNA. 

**Figure 2:**
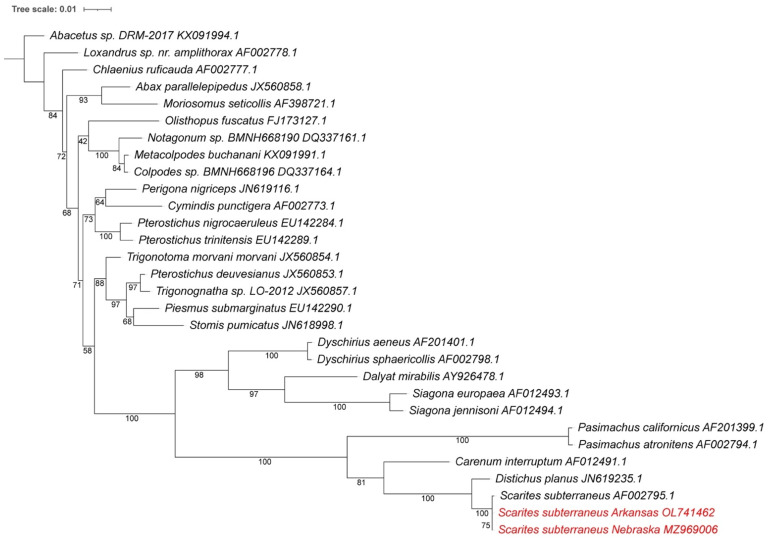
Phylogenetic tree based on 18S rRNA sequences for all available *Scarites* and closest Carabidae. The new isolates are marked in red. Accession numbers are included and *Abecetus* sp. Was added as an outgroup. The phylogenetic tree was generated by using the Maximum Likelihood method and General Time Reversible model [34] within MEGA X [35]. Bootstrap values were inferred from 500 replicates [36]. iTOL was used to draw the phylogenetic trees expressed in the Newick phylogenetic tree format [37].

Figure 2 provides an 18S rRNA-based phylogenetic comparison of the new isolates to the *Scarites* species and other Carabidae sequences in Genbank. This confirms that the isolates indeed belong to the genus *Scarites*, however, they are not identical to the *S. subterraneus* that was previously found [16]. The new 18S rRNA showed seven differences and a 6 bp gap compared to the database sequence, while the 28S rRNA showed 24 differences. In order to confirm these differences and exclude the possibility of sequencing errors in the NGS shotgun sequencing approach, we PCR amplified an 18S rRNA and 28S rRNA fragment using the total extracted DNA and performed Sanger sequencing. The amplified sequences were 100% identical to the same region of the NGS fragments and confirmed the sequence differences with the earlier *subterraneus* sequences from Genbank. These rRNA results indicate that the isolates possibly belong to a different *Scarites* species or a subspecies of *subterraneus*. A deeper genomic comparison was needed to confirm which of these is the case.

### 3.3. Mitogenomes Summary

**Figure 3:**
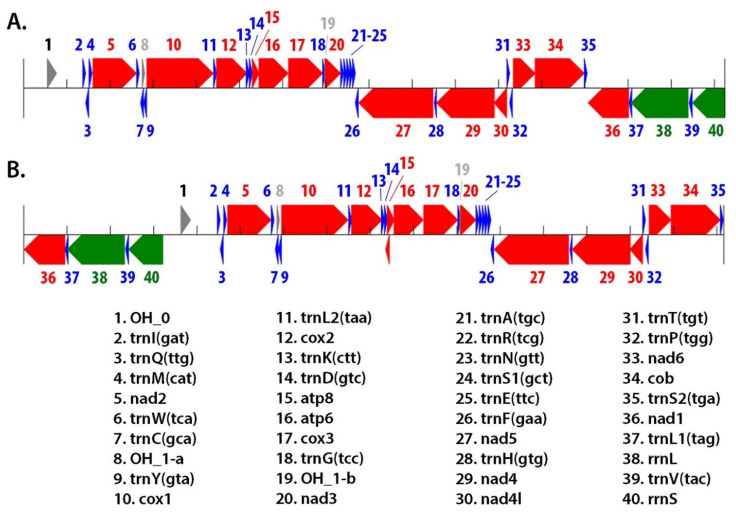
Schematic overview of the gene organization of the annotated mitogenome of *Scarites subterraneus*. (**A**). is the Nebraska isolate mitogenome, and (**B**). is the Arkansas isolate. Mitogenomes were annotated by Mitos2 [28,29]. tRNA genes are in blue, rRNA genes in green, and protein coding genes in red.

The SPAdes assembled 16.2 Mbp contigs from both isolates were used for an automated annotation using MITOS2 [28,29]. This found the Nebraska mitogenome to have a total of 37 genes, with 13 coding sequences (CDS), 2 rRNA genes, and 22 tRNAs (Table S1). The mitogenome organization of *S. subterraneus* is presented in Figure 3A and Supplementary Table S1. There are 23 genes encoded on the positive strand, while the remaining 14 genes are encoded on the negative strand (Table S1 and Figure 3). As seen in other Caribidae mitogenomes, this genome is compact with several overlaps identified in 15 junctions. The overall mitogenome sequence had an average nucleotide composition of A = 41%, T = 38%, C = 13%, and G = 8%. This high AT percentage (79%) is similar to what has been seen in other Carabidae mitogenomes [22]. A very similar genomic organization was found for the Arkansas species (Table S1 and Figure 3B), with an identical total of 37 genes, with 13 coding sequences (CDS), 2 rRNA genes, and 22 tRNAs. This mitogenome had an average nucleotide composition of A = 40%, T = 40%, C = 12%, and G = 8% for the Arkansas species. The overall nucleotide identity between the two mitogenomes was found to be 98.8%, which indicates that they indeed belong to the same species. The Arkansas genome was found to be circular and complete, with the NADH dehydrogenase subunit 1 complete gene (*nad1*) overlapping on both ends of the contig (66bp overlap). The mitogenome from the Nebraska species is also complete and circular with a 72 bp overlap in the untranslated region between the small ribosomal gene rrnS and OH_0 (origin of heavy strand replication) regions. 

Eleven of the protein coding sequences are on the positive strand, while the other three (*nad5*, *nad4*, and *nad1*) are on the negative strand. All protein coding genes start with the typical ATN codons, except for *cox*1, which was initiated with an unconventional TCG start codon. Unconventional start codons for *cox*1 have been observed in other insect species as well [22,40,41]. All protein coding sequences use either TAA or TAG stop codons. In both genomes, the *cox*2 gene ends in a T, and the AA is presumably added by A-tailing as occasionally seen in mitogenomes of other animals. 

The 22 tRNA genes ranged in length from 52 (trnH) to 72 bp (trnV) and are the same in both mitogenomes. Fourteen tRNA genes are located on the positive strand, while the remaining eight are on the negative strand. All tRNAs can be folded into the typical cloverleaf secondary structure, except for trnS1 and trnH, in which one of the arms is replaced by a simple loop. The large ribosomal gene (rrnL) is 1324 bp in length in both mitogenomes, while the small ribosomal gene is 754 bp long in the Nebraska species and 789 bp in the Arkansas species. 

### 3.4. Mitogenome Phylogenetic Analysis

**Figure 4:**
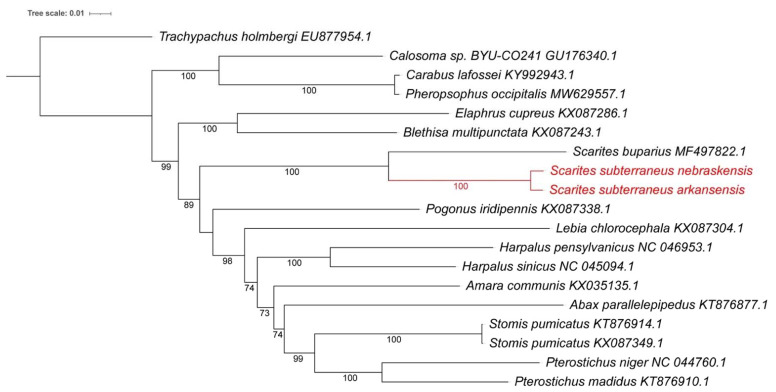
Phylogenetic tree of the mitogenome sequences for all available *Carabidae* (with >85% identity to *Scarites subterraneus*). The two new mitogenomes are marked in red. *Trachypachus holmbergi* was added as an outgroup and belongs to the related *Trachypachidae* family. Accession numbers are included. The phylogenetic tree was generated by using the Maximum Likelihood method and General Time Reversible model [34] within MEGA X [35]. Bootstrap values were inferred from 500 replicates [36]. iTOL was used to draw the phylogenetic trees expressed in the Newick phylogenetic tree format [37].

A genome-based phylogenetic analysis of the closest Coleoptera mitogenomes (Figure 4) showed the three *Scarites* mitogenomes (*buparius*, and the two *subterrraneus* from this study) to be in a separate clade, consistent with them being a separate genus in the Carabidae family. The closest species used for this analysis were selected as having >85% identity to our *Scarites* isolates. It has been suggested to recognize genomes with average nucleotide identity (ANI) >95% as belonging to the same species [38], while genomes with ANI <90% would be recognized in most cases as separate species. Those with values between 90 and 95% identity may be argued either way depending on other properties. ANI has been found to be more precise in the differentiation of closely related species as compared to the use of 18S or 16S rRNA gene sequences [38]. A pairwise comparison of the Nebraksa *Scarites* mitogenome and S. *buparius*, showed them to have an ANI of 90.0%, while the Arkansas *Scarites* and *S. buparius* mitogenomes have 88.9% ANI. Since the two new isolates have an ANI of 98.8%, it is clear that, based on comparative ANI and the mitogenomic phylogenetic tree comparison, the Arkansas and Nebraska isolates belong to the same species that can be distinguished from the *S. buparius* species. The closest genera based on the phylogenetic analysis (Figure 4) appear to be *Blethisa* and *Elaphrus*, with a more distant relationship to *Calosoma*, *Pherosophus,* and *Carabus*. This mitogenome placement of the three *Scarites* mitogenomes within the Carabidae family is consistent with previous genus designations based on physical characteristics or 18S rRNA comparison. 

**Figure 5:**
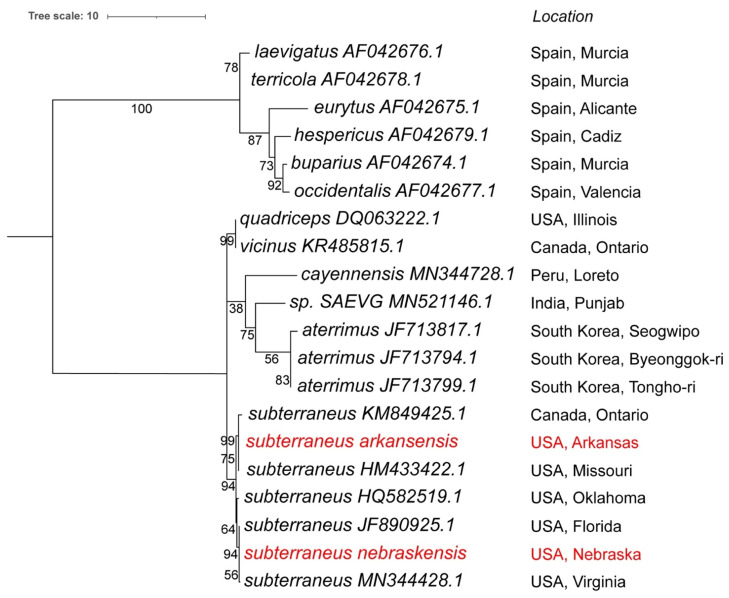
Phylogenetic tree of the known Cox1 gene sequences from all the *Scarites* species. Species names and accession numbers are included. The phylogenetic tree was generated by using the Maximum Likelihood method and General Time Reversible model [34] within MEGA X [35]. Bootstrap values were inferred from 500 replicates [36]. iTOL was used to draw the phylogenetic trees expressed in the Newick phylogenetic tree format [37]. Tree was rooted at midpoint. *Scarites subterraneus nebraskensis* and *arkansensis* are in red.

No other *Scarites* mitogenomes are currently available in Genbank, which limits further taxonomic mitogenome-based analysis at the species level. However, there are 18 different Cox1 gene sequences available for *Scarites* in Genbank. Although no phylogenetic comparison of these has been published, five of these are designated to *S. subterraneus*, three to *S. aterrimus*, and the rest have individual species designations. We used the Cox1 gene from our *Scarites* mitogenomes to perform a phylogenetic comparison within the *Scarites* genus. Figure 5 shows that all the *subterraneus* species, including our two new isolates, form a unique clade on the phylogenetic tree, and therefore all rightfully belong to the same species, consistent with the ANI and whole mitogenome comparisons. The closest relatives are *S. vicinus* and *S. quadriceps*, and more distant is *S. aterrimus*, which is consistent with the identification key description, based on morphological characteristics. This confirms that our isolates indeed belong to *S. subterraneus*, and are clearly only distantly related to *S. buparius*, which belongs to a separate clade on this phylogenetic tree (Figure 5). 

Differences in the rRNA and Cox1 gene sequences indicated that the new isolates are different subspecies of what is currently in the database for *S. subterraneus*. To distinguish the molecular data (mitochondrial genome, 18S and 28S rRNA, and Cox1 gene), we propose the novel subspecies names ‘*nebraskensis*’ and ‘*arkansensis*’, based on the geographical location of these isolates. The mitogenome sequences were deposited to NCBI Genbank as belonging to *Scarites subterraneus* ssp. *Nebraskensis* (accession number: OK032609) and *Scarites subterraneus* ssp. *Arkansensis* (accession number: OL872182).

The Cox1-based phylogenetic analysis is in agreement with the geographical distribution in the genus. As can be seen from Figure 5, the species isolated from the Mediterranean area form a separate clade on the tree (that includes *S. buparius*), clearly distinct from the American and Asian species. The North American *S. subterraneus*, *S. quadriceps,* and *S. vicinus* are evolutionarily closer to each other than to the South American *S. cayennensis* and the South Korean *S. aterrimus* species. However, *S. cayennensis* has the weakest support value on this tree and its exact evolutionary placement may need further study. An earlier phylogenetic study, based on COI sequences and karyotyping analysis, which only compared the species from the West Mediterranean Basin, showed a similar distribution as the upper clade in Figure 5 [42]. In both analyses, the greatest divergence in the Mediterranean clade can be seen between *S. eurytus* and *S. laevigatus*, while *S. laevigatus* and *S. terricola* show the lowest divergence. This comparison is now expanded to include *Scarites* from a wider geographical area, which makes it clear that the species in this genus have evolutionarily adapted to the climatic and geological events at each of the geographic locations. With increasing access and use of whole genome and targeted sequencing, the position of some of these species (for example, *S. cayennensis* possibly in comparison to other South American species) will undoubtedly be further refined.

### 3.5. Microbial Analysis

Although microbial analysis was not the premise of this analysis, since we used the entire genomic DNA extraction and did not select for any targeted sequencing, we had the opportunity to also analyze these datasets for potential microbial and viral signatures that could be an indication of endosymbionts of these *Scarites* specimens. Even though beetle gut insides were removed and the samples were rinsed with ethanol, the microbial fraction still represented 10% and 29% of the total reads in the Nebraska and Arkansas datasets. A Kraken2 metagenomic analysis showed that the Arkansas samples had the most microbial reads and diversity; however, this is likely due to the larger sequencing depth of this sample. The fact that this sample had only been preserved for 2 months, as compared to the Nebraska samples that had been stored in ethanol for over a year, could also be a contributing factor. 

The Arkansas *Scarites* sample mainly contained the following three bacterial phyla: Proteobacteria (49%), Bacteroides (25%), and Terrabacteria (23%). The highest represented genera were *Providencia* (36% of bacteria), *Myroides* (18% of bacteria), and *Spiroplasma* (5% of bacteria). 

*Providencia* is a known pathogenic bacteria isolated from insects, mainly from fruitflies and nematode larvae [43,44,45], where it often results in insect mortality. This is the first time *Providencia* is reported to be associated with *Scarites,* or Coleoptera in general, but such association may not be too surprising given the pathogen’s wide host variety. *Myroides* belongs to the Flavobacteria and has known human and fish pathogenic strains [46,47,48]. Several multiantibiotic resistant strains have been found in flies [49,50], however, little is currently known about its potential role in beetles. 

Interestingly, *Spiroplasma* has been found as an endosymbiont in other beetle species, where it is presumably involved in host defense against nematodes and parasitoids through ribose-inactivating proteins (RIPs) [51], or contrariwise act as a reproductive parasite itself [52]. Recently, it has been suggested to be involved in cold adaptation of high elevation *Nebria* species [53]. Until now, no reports had been made about *Spiroplasma* endosymbionts in *Scarites*, but this finding is consistent with the suggestion by Weng et al. [53], that *Spiroplasma* might be widespread in the Carabidae. 

Besides bacteria, the only viral signature found in the assembled *Scarites* Arkansas genome was for *Nucleocytoviricota* (50% *Chrysochromulina ericina* (*Mimiviridae* family); 50% BeAn 58,058 (*Poxviridae* family)), which are nucleocytoplasmic large DNA viruses [54]. The identified viral species belong to the giant viruses that typically infect protozoa, invertebrates and eukaryotic algae [54,55]. This is the first report of such giant virus signature associated with *Scarites* beetles, however, further research will be needed to show whether *Scarites* is a host for these viruses. 

## 4. Conclusions

Using total genomic DNA extraction, followed by whole genome library preparation and Illumina-based short-read sequencing, we were able to obtain a full mitogenome sequence, in addition to the 18S and 28S rRNA sequences, from *Scarites* isolates from both Nebraska (US) and Arkansas (US). The designed protocol was relative straight-forward, without the need for a separate mitochondrial DNA isolation (only using genomic DNA from crushed beetle tissue), and only using Illumina short-read data and available web-based bioinformatics. The short-read assembled mitogenome analysis was found to be accurate and sufficient to perform a taxonomic comparison down to the subspecies level. Subspecies could be distinguished based on their mitogenomic differences (mitogenomic ANI values) but had identical 18S rRNA sequences. In addition, this broad scale sequencing allowed us to also identify bacterial and viral species, that could form potential endosymbiotic or pathogenic associations with the *Scarites* specimen. 

Genetic and mitogenomic analysis found our species to belong to *S. subterraneus*, but with different 18S, 28S, and Cox1 sequences as compared to *Scarites* specimens that are currently available. We therefore designated these isolates as *Scarites subterraneus* ssp. *nebraskensis* and *Scarites subterraneus* ssp. *arkansensis*. This is the first report of a mitogenome of any *Scarites subterraneus*. The closest available mitogenome is from *S. buparius*, with 88–90% ANI. The mitogenome-based phylogenetic analysis allowed for a comprehensive overview of the *Carabidae*, while the Cox1 gene and ANI comparison generated a fine-tuned placement of the new isolates within the *Scarites* genus. The molecular phylogenetic data is in good agreement with the geographical distribution and morphological characteristics of the species in this genus. 

Given the relative simplicity and general availability of the methods used, applied to preserved specimen, and the use of only web-based bioinformatic tools, we believe this method allows for a more general broader scale application for taxonomic analysis of Coleoptera specimen and other invertebrates. A streamlined method as presented here to obtain the mitogenome (including *cox*1) and 18S/28S rRNA data is beneficial to current DNA barcoding practices [56], which are typically *cox*1-based in insects. The goal of DNA barcoding is to build a global library that can be used for biodiversity studies or verification in collection management systems. This practice certainly requires logistic efficiency and depends on the global availability of molecular data for comparison [56]. It remains to be seen whether the presented method can also be used based on DNA extraction methods that keep the specimen intact [57], however, if sufficient mitochondrial or nuclear DNA is obtained, the same short-read sequencing and data analysis approach should theoretically be feasible.

## Data Availability

The mitochondrial genome sequences were deposited to NCBI Genbank as belonging to *Scarites subterraneus* ssp. *nebraskensis* (accession number: OK032609) and *Scarites subterraneus* ssp. *arkansensis* (accession number: OL872182). The 18S rRNA sequences were also deposited to NCBI Genbank with the following accession numbers: MZ969006 (*Scarites subterraneus* ssp. *nebraskensis* (*=strain Kyndtius*)) and OL741462 (*Scarites subterraneus* ssp. *arkansensis*).

## Supplementary Materials

The following are available online at https://www.mdpi.com/article/10.3390/insects13020190/s1, Table S1: Organization of the *Scarites subterraneus* mitogenome. A. *Scarites subterraneus* ssp. *nebraskensis*. B. *Scarites subterraneus* ssp. *arkansensis*. Mitogenomes were annotated using Mitos2.
