# Supplementary material for: Illumina Short-Read Sequencing of the Mitogenomes of Novel Scarites subterraneus Isolates Allows for Taxonomic Refinement of the Genus Scarites Fabricius 1775, within the Carabidae Family
Source: Insects. 2022 Feb 11;13(2):190. doi: 10.3390/insects13020190 (PMC8874491; doi:10.3390/insects13020190)
Supplement: Supplementary file 1 [file insects-13-00190-s001.zip › insects-1548678-supplementary.pdf]

Table S1. Organization of the *Scarites subterraneus* mitogenome. A. *Scarites subterraneus* ssp. *nebraskensis*. B. *Scarites subterraneus* ssp. *arkansensis*. Mitogenomes were annotated using Mitos2.

Scarites. sp nebraskensis				
**Name**	**Start**	**Stop**	**Strand**	**Length**
OH_0	550	764	+	215
trnI(gat)	1371	1436	+	66
trnQ(ttg)	1439	1507	-	69
trnM(cat)	1517	1585	+	69
nad2	1607	2614	+	1008
trnW(tca)	2617	2680	+	64
trnC(gca)	2710	2777	-	68
OH_1-a	2748	2804	+	57
trnY(gta)	2783	2850	-	68
cox1	2856	4391	+	1536
trnL2(taa)	4401	4466	+	66
cox2	4468	5175	+	708
trnK(ctt)	5156	5226	+	71
trnD(gtc)	5227	5293	+	67
atp8_0	5294	5455	+	162
atp6	5449	6126	+	678
cox3	6136	6924	+	789
trnG(tcc)	6924	6989	+	66
OH_1-b	6971	7036	+	66
nad3	6990	7343	+	354
trnA(tgc)	7342	7406	+	65
trnR(tcg)	7415	7481	+	67
trnN(gtt)	7486	7551	+	66
trnS1(gct)	7552	7618	+	67
trnE(ttc)	7619	7686	+	68
trnF(gaa)	7685	7751	-	67
nad5	7762	9480	-	1719
trnH(gtg)	9512	9563	-	52
nad4	9563	10903	-	1341
nad4l	10897	11190	-	294
trnT(tgt)	11193	11257	+	65
trnP(tgg)	11258	11322	-	65
nad6	11343	11849	+	507
cob	11849	12985	+	1137
trnS2(tga)	12985	13052	+	68
nad1	13074	14015	-	942
trnL1(tag)	14025	14089	-	65
rrnL	14067	15390	-	1324
trnV(tac)	15416	15487	-	72
rrnS	15488	16241	-	754

Scarites sp. arkansensis				
**Name**	**Start**	**Stop**	**Strand**	**Length**
nad1	10	951	-	942
trnL1(tag)	961	1025	-	65
rrnL	1003	2326	-	1324
trnV(tac)	2351	2422	-	72
rrnS	2423	3211	-	789
OH_0	3626	3854	+	229
trnI(gat)	4470	4535	+	66
trnQ(ttg)	4538	4606	-	69
trnM(cat)	4616	4684	+	69
nad2	4706	5713	+	1008
trnW(tca)	5716	5779	+	64
trnC(gca)	5809	5876	-	68
OH_1-a	5847	5903	+	57
trnY(gta)	5882	5949	-	68
cox1	5955	7490	+	1536
trnL2(taa)	7500	7565	+	66
cox2	7567	8274	+	708
trnK(ctt)	8255	8325	+	71
trnD(gtc)	8326	8392	+	67
atp8_0	8393	8554	+	162
atp6	8548	9225	+	678
cox3	9235	10023	+	789
trnG(tcc)	10023	10088	+	66
OH_1-b	10070	10135	+	66
nad3	10089	10442	+	354
trnA(tgc)	10441	10505	+	65
trnR(tcg)	10514	10580	+	67
trnN(gtt)	10585	10650	+	66
trnS1(gct)	10651	10717	+	67
trnE(ttc)	10718	10785	+	68
trnF(gaa)	10784	10850	-	67
nad5	10861	12579	-	1719
trnH(gtg)	12611	12662	-	52
nad4	12662	14002	-	1341
nad4l	13996	14289	-	294
trnT(tgt)	14292	14356	+	65
trnP(tgg)	14357	14421	-	65
nad6	14442	14948	+	507
cob	14948	16084	+	1137
trnS2(tga)	16084	16151	+	68
